# Acupuncture combined with Tuina therapy for the treatment of anisometropic amblyopia and track visual cortex activation status: A case report

**DOI:** 10.1097/MD.0000000000046551

**Published:** 2025-12-19

**Authors:** Xinyu Zhao, Ailing Bi, Xiuzhen Lu, Hui Wang, Xiaotong Wu, Jianfeng Wu, Hongsheng Bi

**Affiliations:** a Shandong University of Traditional Chinese Medicine, Jinan, Shandong Province, P.R. China; b Shandong Academy of Eye Disease Prevention and Therapy, Shandong Key Laboratory of Integrated Traditional Chinese and Western Medicine for Prevention and Therapy of Ocular Diseases, Shandong Provincial Clinical Medical Research Center of Optometry and Children Visual Impairment Prevention and Control, Shandong Engineering Technology Research Center of Visual Intelligence, Shandong Institute of Children Health and Myopia Prevention and Control, Jinan, Shandong Province, P.R. China; c Affiliated Eye Hospital of Shandong University of Traditional Chinese Medicine, Jinan, Shandong Province, P.R. China.

**Keywords:** acupuncture, amblyopia, case report, mechanisms, Tuina

## Abstract

**Rationale:**

Amblyopia is a common pediatric neurodevelopmental disorder of vision, necessitating early intervention to improve visual acuity. We report a case of a child with anisometropic amblyopic whose best corrected visual acuity of the right eye (OD, amblyopic eye) remained stable at 0.6 LogMAR despite 10 months of conventional occlusion therapy. We further evaluated the therapy’s efficacy using functional near-infrared spectroscopy (fNIRS) to monitor visual cortex activation.

**Patient concerns:**

A 6-year-old boy with anisometropic amblyopia came to seek Chinese medicine treatment in June 2020. Before 2020, the child had undergone 2 years of binocular optical correction and relatively good occlusion treatment. After a 10-month period of visual plateau (BCVA stable at 0.6 LogMAR) despite conventional treatment, we tried the treatment of acupuncture combined with Tuina therapy.

**Diagnoses:**

A case of anisometropic amblyopia refractory to conventional occlusion therapy.

**Interventions:**

The patient received acupuncture combined with Tuina therapy (Chinese therapeutic massage).

**Outcomes:**

After 10 sessions of acupuncture combined with Tuina therapy, the OD best corrected visual acuity improved to 0.2 LogMAR, representing a 0.4 LogMAR gain.

**Lessons:**

Acupuncture combined with Tuina therapy was associated with improved visual acuity in this single-case of amblyopia, suggesting potential as a new treatment approach for those who exhibit poor compliance or inadequate response to traditional therapies. However, as a case report, these findings are preliminary and require validation in larger controlled studies. Additionally, the study proposes that fNIRS can serve as a convenient and effective technique for monitoring the impact of acupuncture and Tuina on the visual cortex, providing an evaluation metric for future integrated eye-brain treatments.

## 1. Introduction

Amblyopia, a neurodevelopmental disorder arising from abnormal visual experiences during the critical period of visual maturation, manifests as reduced best corrected visual acuity (BCVA) in 1 or both eyes without detectable organic lesions.^[1]^ Recent studies have shown that the total prevalence of amblyopia is about 1.44%.^[2]^ Despite the widespread application of conventional therapies such as optical correction and occlusion, suboptimal efficacy persists in pediatric populations due to poor treatment compliance. In China and around the world, acupuncture and Tuina (Chinese therapeutic massage) have been used in the treatment of eye diseases which includes amblyopia. Several studies have reported their efficacy; however, the specific mechanism unclear.^[3–5]^ It is noteworthy that the evidence for acupuncture is more substantiated by modern clinical trials compared to that for Tuina. This single-case report presents a child with refractory anisometropic amblyopia who exhibited apparent visual improvement following combined acupuncture and Tuina therapy, though the generalizability of these findings is limited by the study design. Using functional near-infrared spectroscopy (fNIRS), we investigated treatment-induced neurovascular changes in the visual cortex, exploring potential mechanisms through which these complementary therapies may enhance visual function via modulation of cortical hemodynamic responses and neural activity.

## 2. Case description

**Figure 1.**
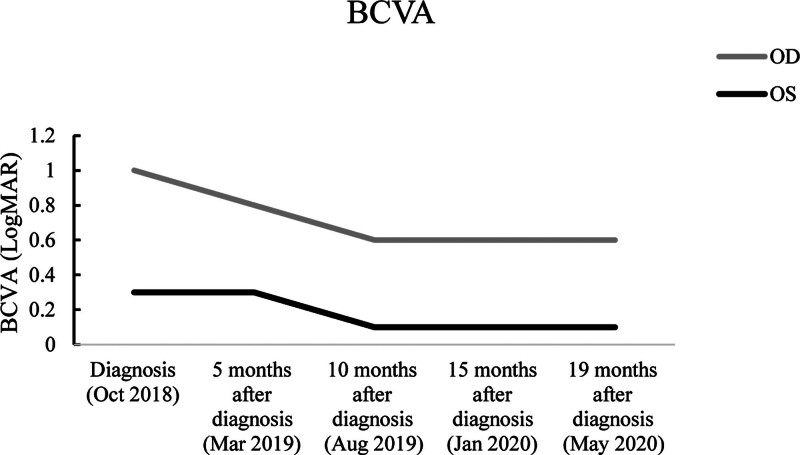
Change in BCVA (LogMAR) of the OD and OS from first diagnosis of anisometropic amblyopia to pretreatment with acupuncture and Tuina therapy. Note: The x-axis represents the time points (October 2018 to May 2020), and the y-axis represents BCVA (LogMAR). The trajectory shows that OD (the amblyopic eye) improved from 1.0 LogMAR to 0.6 LogMAR over a 10-month period, followed by a 10-month plateau phase (August 2019 to May 2020). OS remained stable after initial improvement. Lower LogMAR values denote better visual acuity. BCVA = best corrected visual acuity, OD = right eye, OS = left eye.

A 6-years-old male with anisometropic amblyopia (diagnosed October 2018), during the 20-month conventional treatment, the patient underwent optical correction with prescribed glasses (updated every 3–6 months based on refraction change) and daily occlusion of the non-amblyopic eye (left eye) for 6 hours, as confirmed by parental logs and monthly clinician assessments (adherence rate > 90%). Despite consistent intervention, the amblyopic eye (right eye) showed no BCVA improvement from August 2019 to May 2020 (LogMAR remained 0.6; Fig. 1 and Table 1). This sustained lack of improvement, despite reportedly good adherence to conventional care, supports the classification of this case as refractory amblyopia. The same technician performed all fNIRS assessments at standardized time points (30 minutes prior to each subsequent acupuncture and Tuina session; for example, the effects of the second treatment were measured before the third treatment). This protocol ensured that the measurements reflected the cumulative therapeutic effects rather than interference signals generated by transient needling stimulation.

**Table 1:** BCVA and refraction in the patient from initial diagnosis to prior to acupuncture combined with Tuina therapy.

Timepoint	Refraction		BCVA	
	OD (Diopter)	OS (Diopter)	OD (LogMAR)	OS (LogMAR)
Diagnosis (October 2018)	+6.75	+4.75	1.0	0.3
5 months after diagnosis (March 2019)	+6.00	+4.25	0.8	0.3
10 months after diagnosis (August 2019)	+5.50	+3.50	0.6	0.1
15 mo after diagnosis (January 2020)	+5.00	+3.25	0.6	0.1
19 mo after diagnosis (May 2020)	+4.25	+2.75	0.6	0.1

Date include refractive status (diopter) and BCVA (LogMAR) of the OD and OS at key time points: from initial diagnosis (October 2018) to 1 mo before the initiation of acupuncture-Tuina therapy (May 2020). The trajectory highlights the amblyopic eye (OD) improvement followed by a 10-mo plateau (Augest 2019 to May 2020), supporting refractory status to conventional treatment. Lower LogMAR values denote better visual acuity.

BCVA = best corrected visual acuity, OD = right eye, OS = left eye.

This study was approved by the ethics committee of the Affiliated Eye Hospital of Shandong University of Traditional Chinese Medicine (Approval number [HEC-KS-2020013KY02]). The study protocol conforms to the ethical guidelines of the Declaration of Helsinki, and informed consent was obtained from the patient’s guardian prior to participation.

Following the initial 10-session intervention in 2020, the patient underwent regular long-term follow-up assessments to monitor the stability of visual outcomes. As shown in Table 2, the BCVA of the amblyopic right eye (OD) was maintained at 0.1 LogMAR from March 2022 through November 2024, demonstrating the durability of the therapeutic effect achieved through acupuncture and Tuina therapy. The non-amblyopic left eye (OS) consistently maintained excellent vision (0.0 LogMAR) throughout the follow-up period.

**Table 2:** Long-term follow-up of refraction and BCVA after acupuncture combined with Tuina therapy.

Timepoint	Refraction (Diopter)		BCVA (LogMAR)	
	OD	OS	OD	OS
October 2020	+3.125	+1.875	0.2	0.0
April 2021	+3.375	+1.625	0.2	0.0
September 2021	+3.125	+1.000	0.2	0.0
Novomber 2021	+3.125	+1.375	0.2	0.0
March 2022	+3.375	+1.625	0.1	0.0
February 2023	+3.250	+1.625	0.1	0.0
January 2024	+3.125	+1.500	0.1	0.0
November 2024	+3.125	+1.125	0.1	0.0

Note: This table presents the refractive status (diopter) and BCVA (LogMAR) of the OD and OS during the long-term follow-up period after the completion of the acupuncture-Tuina intervention. The data show that the visual acuity improvement in the amblyopic eye (OD) was maintained at 0.1 LogMAR from March 2022 onward, demonstrating the long-term stability of the treatment effect. Lower LogMAR values denote better visual acuity.

BCVA = best corrected visual acuity, OD = right eye, OS = left eye.

## 3. Therapeutic intervention

**Figure 2.**
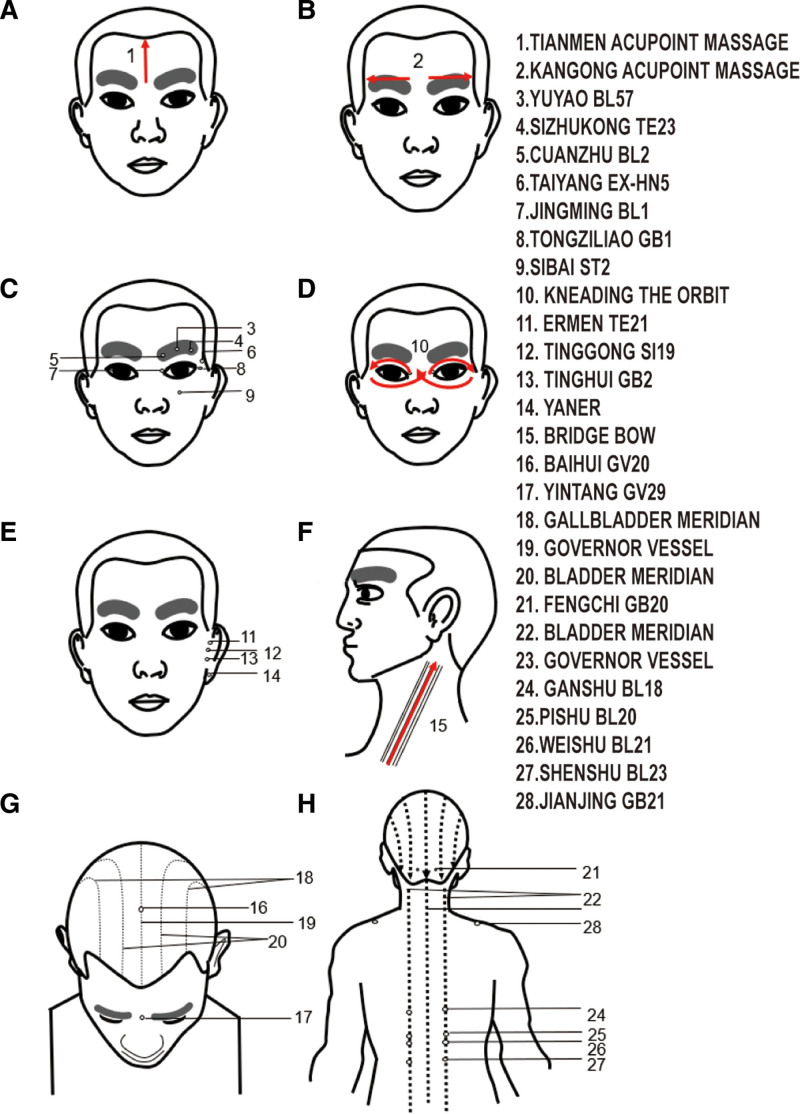
Standardized Tuina acupoint localization and manipulation protocol. The procedure integrates: (1) head-face relaxation (Tianmen, Kangong, Taiyang) to soothe muscles; (2) periocular stimulation (Jingming BL1, Cuanzhu BL2, Yuyao EX-HN4, etc) to target local acupoints; (3) Meridian regulation (Governor Vessel, Bladder, Gallbladder Meridians) for systemic balance; (4) Auxiliary modulation (auricular points, Bridge bow) to reinforce efficacy. Arrows indicate manipulation direction. All steps were performed bilaterally. In the schematic, each module is clearly labeled.

**Figure 3.**
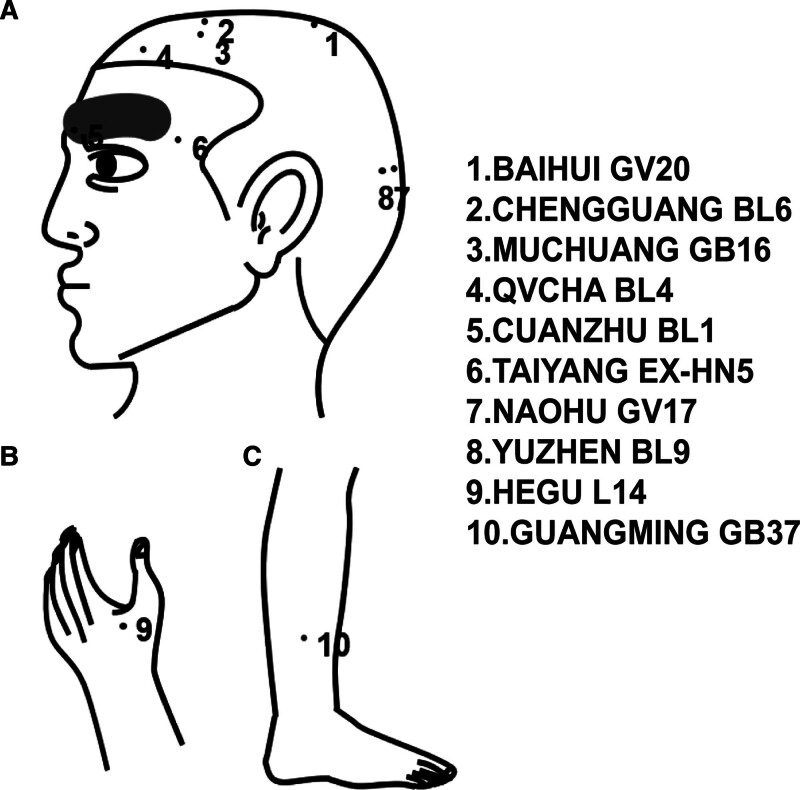
Acupuncture point schema and needling parameters. Anatomical distribution of acupoints selected for amblyopia treatment: Cuanzhu (BL2), Taiyang (EX-HN5), Baihui (GV20), Qvcha (BL4), Muchuang (GB16), Chengguang (BL6), Naohu (GV17), Yuzhen (BL9), Hegu (LI4), and Guangming (GB37). Periocular/cranial points (A) were superficially needled (0.3 inches, flat insertion with gentle rotation); distal points (B and C) were inserted to 0.5 inches. Needles (0.20 × 0.25 mm, disposable sterile) were retained for 15 min. Point localization followed WHO standards.

Before the acupuncture and Tuina therapy, we conducted a comprehensive assessment of the patient’s condition, explained the treatment protocol, and ensured the informed consent of the patient. Management was performed by a physician with extensive clinical experience. The treatment regimen comprised 3 components: refractive correction with prescribed glasses; daily occlusion therapy (6 hours); and combined acupuncture and Tuina therapy, initiated concurrently with optical correction. Treatments were administered each morning, with 1 course consisting of 10 sessions (5 sessions/week). Tuina preceded acupuncture and was performed by an experienced clinician following a standardized protocol: The patient was seated in a comfortable position. Each step was performed bilaterally with moderate, consistent pressure deemed tolerable by the child. The specific sequence and parameters were as follows: first: pushing (thumb manipulation) from Tianmen (midpoint between eyebrows hairline) to the hairline, and arc-pushing (Kangong) from the midpoint of the eyebrows to the temples (30 repetitions each), until mild erythema and a local sensation of warmth were observed. Second: kneading (circular motion) of Taiyang (EX-HN5, in the temporal fossa) using the middle finger (30 repetitions). Third: Pressing and circular-rubbing of the periocular acupoints Jingming (BL1, medial canthus), Cuanzhu (BL2, eyebrow medial end), Yuyao (EX-HN4, midpoint of the eyebrow), Sizhukong (TE23, eyebrow lateral end), Tongziliao (GB1, lateral canthus), and Sibai (ST2, infraorbital foramen) using the index finger (30 repetitions per acupoint). Fourth: light kneading with the thumb in a figure-of-eight (“∞”) pattern along the orbital rim (superior and inferior) for 2 minutes. Fifth: Steady pressure applied to the auricular points Tinggong (SI19, anterior to the tragus), Tinghui (GB2, posterior to the tragus), Ermen (TE21, superior to the tragus), and the central earlobe (30 repetitions each). Sixth: Pinching and gently lifting the tissue of the “Bridge bow” (the area between the index finger and thumb on the back of the hand) using the thumb, index, and middle fingers for 2 minutes. Seventh: kneading from Yintang (GV29, between the eyebrows) along the midline to Baihui (GV20, vertex of the head), followed by light Meridian manipulation (stroking) along the Governor Vessel, Bladder Meridian (bilateral to the spine), and Gallbladder Meridian (lateral head), concluding with kneading of Fengchi (GB20, base of the skull) (20 repetitions). Eighth: Dorsal Meridian dredging using palm-pushing along the Governor Vessel and bilateral Bladder Meridian channels on the back, with focused thumb-pressure on the back-shu points Ganshu (BL18), Pishu (BL20), Weishu (BL21), Shenshu (BL23), and Jianjing (GB21) (30 repetitions per point) (Fig. 2). The entire Tuina session lasted approximately 20 minutes. Acupuncture procedure: sterile disposable acupuncture needles (size: 0.20 mm in diameter × 25 mm in length, Zhongyan Taihe, China) were used. After standard skin disinfection with 75% alcohol, needles were inserted at the following acupoints: Cuanzhu (BL2), Taiyang (EX-HN5), Baihui (GV20), Qucha (BL4), Muchuang (GB16), Chengguang (BL6), Naohu (GV17), Yuzhen (BL9), Hegu (LI4), and Guangming (GB37). Point localization followed the WHO Standard Acupuncture Point Locations. For periocular (BL2, EX-HN5) and cranial points (GV20, BL4, GB16, BL6, GV17, BL9), needles were inserted subcutaneously to a depth of approximately 0.3 cun (approx. 7.5 mm) using a transverse (flat) insertion technique, followed by gentle, even rotation (tonifying and reducing method) to achieve “De Qi” sensation (a sensation of soreness, numbness, or distension reported by the patient) where feasible and tolerable. For distal points on the limbs (LI4, GB37), needles were inserted perpendicularly to a depth of approximately 0.5 cun (approx. 12.5 mm) and manually stimulated to elicit “De Qi.” All needles were retained for 15 minutes. During retention, no additional manual stimulation was applied (Fig. 3).

To minimize potential bias, all therapeutic interventions were administered by the same licensed acupuncturist (>10 years of acupuncture and Tuina/massage experience) adhering to standardized protocol. Treatments were consistently delivered between 8:00 and 10:00 am to control for circadian variations. Objective monitoring of treatment adherence included: daily detailed logs maintained by the parents, noting the occlusion duration and whether the daily 6-hour requirement was met; before each treatment session, research assistants not involved in the treatment conducted verbal inquiries to verify details of the child’s previous treatment (e.g., start time of acupuncture, compliance during acupuncture and Tuina, etc) and cross-checked these with the log records. However, it is important to note that the primary reliance on parental logs, without the use of electronic monitoring devices (e.g., light-sensing occlusion patches), represents a limitation in the objective verification of occlusion adherence.

## 4. Clinical outcomes

**Figure 4.**
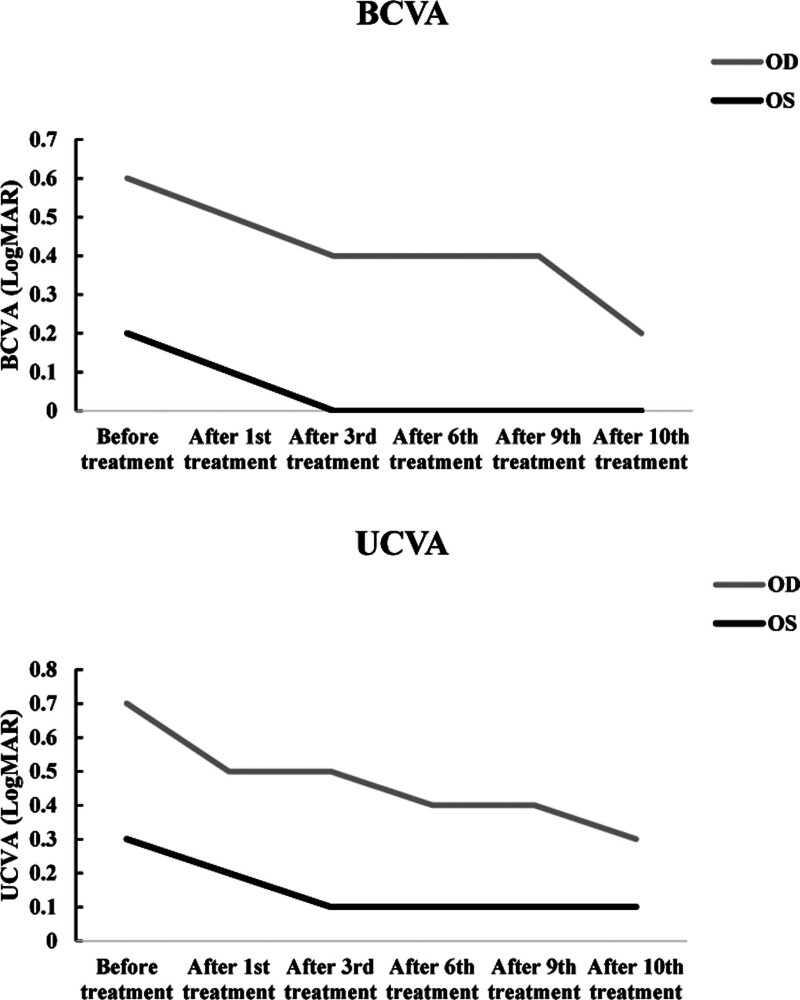
Changes in BCVA (LogMAR) and UCVA (LogMAR) of the OD and OS from before treatment to after the 10th acupuncture-Tuina Treatment session. Note: The x-axis represents the time points (before treatment, after the 1st, 3rd, 6th, 9th, and 10th treatment sessions), and the y-axis represents BCVA and UCVA (LogMAR). (A) shows the trajectory of BCVA: OD (the amblyopic eye) improved progressively from 0.6 LogMAR to 0.2 LogMAR over the 10 treatment sessions, while OS improved from 0.2 LogMAR to 0.0 LogMAR by the 3rd session and remained stable thereafter. (B) shows the trajectory of UCVA: OD improved from 0.7 LogMAR to 0.3 LogMAR, and OS improved from 0.3 LogMAR to 0.1 LogMAR by the 3rd session and remained stable. Lower LogMAR values denote better visual acuity. BCVA = best corrected visual acuity, OD = right eye, OS = left eye, UCVA = uncorrected visual acuity.

We tracked the uncorrected visual acuity (UCVA) and BCVA before and after treatment. The amblyopic eye demonstrated progressive improvement in BCVA from baseline 0.6 LogMAR to 0.2 LogMAR after 10 treatment sessions, with therapeutic gains emerging as early as the first session (0.6 LogMAR→0.5 LogMAR). The non-amblyopic eye maintained excellent baseline BCVA (0.2 LogMAR) that normalized to 0.0 LogMAR by session 3, while UCVA improved from 0.3 LogMAR to 0.1 LogMAR (Table 3 and Fig. 4).

**Table 3:** BCVA and UCVA in the patient during the course of acupuncture combined with Tuina therapy.

	BCVA		UCVA	
	OD (LogMAR)	OS (LogMAR)	OD (LogMAR)	OS (LogMAR)
Before treatment	0.6	0.2	0.7	0.3
After 1^st^ treatment	0.5	0.1	0.5	0.2
After 3^rd^ treatment	0.4	0	0.5	0.1
After 6^th^ treatment	0.4	0	0.4	0.1
After 9^th^ treatment	0.4	0	0.4	0.1
After 10^th^ treatment	0.2	0	0.3	0.1

Date include BCVA and UCVA of the OD and OS, quantified in LogMAR, at baseline (before treatment) and following the 1st, 3rd, 6th, 9th, and 10th acupuncture-Tuina treatment sessions. The data capture progressive improvement in OD (the amblyopic eye, as indicated by poorer baseline vision) and stable visual function in OS, enabling quantitative assessment of treatment efficacy over the intervention course. Lower LogMAR values denote better visual acuity.

BCVA = best corrected visual acuity, OD = right eye, OS = left eye, UCVA = uncorrected visual acuity.

**Figure 5.**
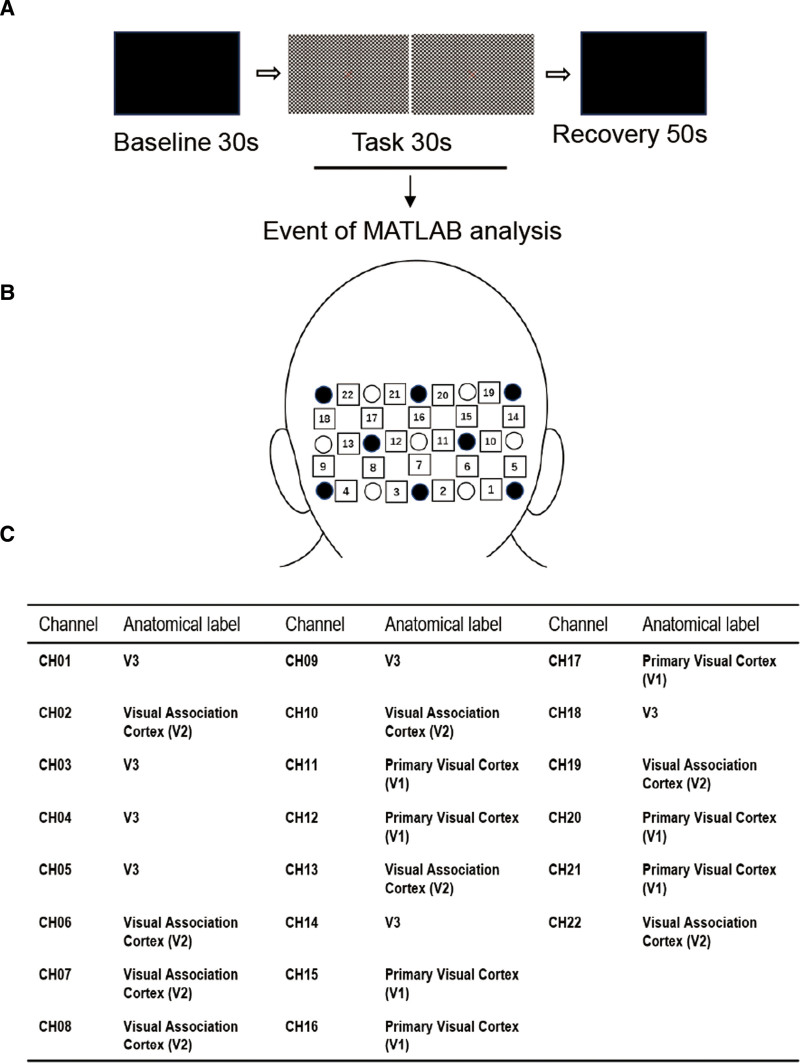
fNIRS experimental design, probe configuration, and cortical mapping. (A) Visual task paradigm: baseline (black screen, 30 s) → task (2 Hz reversing black-white checkerboard, 100% contrast, 15 cpd spatial frequency, 30s) → rest (black screen, 50s). (B) probe array: 15 optodes (8 sources ●, 7 detectors ○) arranged in a 3 × 5 grid centered at the occipital tubercle, forming 22 channels (□). Inter-optode distance: 30 mm. (C) anatomical channel mapping: channels 11/15/20 overlaid right primary visual cortex (V1); channels 12/17/21 overlaid left V1. fNIRS = functional near-infrared spectroscopy.

In order to assess the therapeutic effect of acupuncture and Tuina, especially the impact of acupuncture on the visual cortex of the brain, we employed fNIRS to monitor the brain function before, during and after acupuncture and Tuina therapy. Based on Roland VEP’s vision task, a checkerboard flip visual stimulus for near-infrared brain function detection was designed. The contrast of the black and white checkerboard is 100%, the checkerboard flipping frequency is 2Hz, and the spatial frequency is 15 ‘(Fig. 5A). There were a total of 15 probes for NIRS detection, which were divided into 3 lines, each line containing 5 probes, and the center probe was placed at the occipital tubercle. The anatomical relationship between the probe and the visual cortex was detected by the 3-dimensional (3D) stereotaxic system, and 22 channels were formed by 15 probes. The 3D positioning showed that channels 11, 15 and 20 are located in the right primary visual cortex, while channels 12, 17 and 21 are located in the left primary visual cortex (Fig. 5B and C). Therefore, this study mainly investigated the blood oxygen response of the left and right primary visual cortex.

fNIRS data were acquired using the ETG-4000 system (Hitachi Medical Corporation, Tokyo, Japan). The system utilizes 2 wavelengths of near-infrared light (695 nm and 830 nm). The patient, wearing the custom head probe, was seated in a darkened, quiet room. The probe holder was positioned according to the international 10–20 system, with the bottom row of the 3 × 5 optode grid aligned horizontally, ensuring probe #14 was positioned at the inion. The patient was positioned 1 meter from the stimulus screen with BCVA correction. The stimulus paradigm (Fig. 5A), controlled by E-Prime software (Psychology Software Tools, USA), comprised a single block of: a 30-second initial baseline (black screen), a 30-second task period (full-field, 2Hz reversing black-and-white checkerboard with 100% contrast and 15 cycles per degree spatial frequency, featuring a central red “X” for fixation), and a 50-second rest period (black screen). The total duration of the fNIRS recording per session was 110 seconds. The patient was instructed to maintain postural stability, minimize head movement, and breathe normally while fixating on the central “X” during the task. Device parameters were set to “Stim Measurement” mode. External triggers from the stimulus presentation software were synchronized with the fNIRS data acquisition via a serial port connection (Serial IN, COM1). Real-time hemoglobin concentration change output (Realtime Hb Out) was activated for monitoring. Prior to formal data collection, signal quality for all channels, especially key channels over the primary visual cortex (CH11, CH12, CH15, CH17, CH20, CH21), was verified using the system’s Auto Gain functionality, requiring a stable green indicator for acceptance. fNIRS data were processed using the NIRS_SPM toolbox, which operates within the MATLAB (R2013b) environment and employs the general linear model for statistical analysis.^[6]^ Within the general linear model framework, expressed as Y = Xβ+ε, the measured fNIRS time series data (Y) are modeled as a combination of the predicted hemodynamic response (X), derived by convolving the stimulus paradigm with a hemodynamic response function, and the residual error term (ε). The regression coefficient (β) served as the key parameter, quantifying the magnitude of visual cortex activation in response to the stimulus. Prior to statistical modeling, the raw fNIRS signals underwent preprocessing to mitigate noise from motion artifacts, cardiac pulsation, and instrumental interference. This was achieved through the application of a hemodynamic response function and a discrete cosine transform, as implemented in the NIRS_SPM toolbox.^[6]^

**Figure 6.**
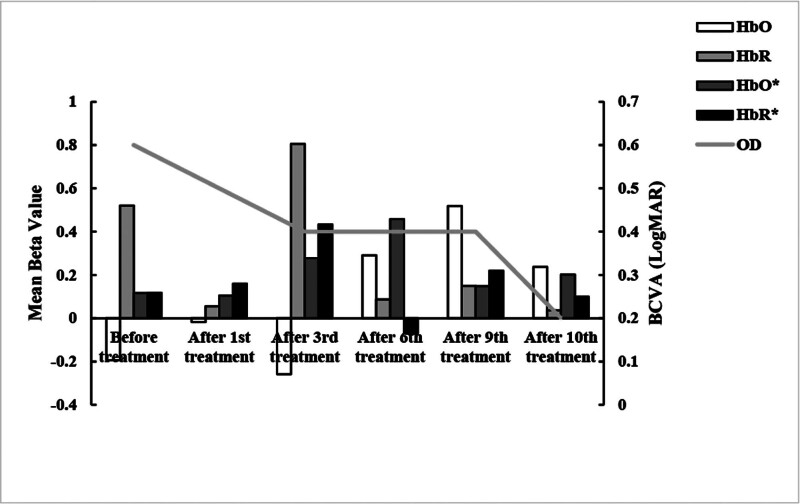
Synchronized improvement in visual cortex activation and clinical visual acuity following combined acupuncture and Tuina therapy. The bar graph (left Y-axis) depicts the mean β-values of HbO and HbR in the visual cortex ipsilateral to the amblyopic eye. Note the progressive increase in HbO and concurrent decrease in HbR, a hemodynamic pattern indicative of enhanced neural activation. The line graph (right Y-axis) tracks the BCVA (LogMAR) of the amblyopic eye (OD), where lower values indicate better vision. Corresponding data for the contralateral visual cortex is shown for comparison. Note: * denotes the contralateral visual cortex. BCVA = best corrected visual acuity, HbO = oxygenated hemoglobin, HbR = deoxygenated hemoglobin, OD = right eye.

To objectively evaluate the treatment’s impact on visual cortex function, we used fNIRS to monitor hemodynamic responses. The fNIRS data revealed a progressive normalization of cortical function specifically in the ipsilateral visual cortex to the amblyopic eye. In contrast, the contralateral visual cortex showed relatively stable hemodynamic responses throughout the treatment, highlighting the specificity of the intervention’s effect. As illustrated in Figure 6, the oxygenated hemoglobin (HbO) β values in the ipsilateral cortex showed a general increasing trend over the treatment course, concurrent with an improvement in the BCVA of the amblyopic eye (OD). Specifically, the ipsilateral HbO β value increased from a negative baseline value (-0.19) to positive values posttreatment (peaking at 0.52 after the 9th session), while the BCVA improved from 0.6 LogMAR to 0.2 LogMAR. This synchronous improvement in ipsilateral cortical activation and visual acuity suggests a positive effect of the combined therapy on neurovisual processing.

## 5. Discussion

This case report explores the potential neurophysiological mechanisms of a combined acupuncture and Tuina therapy for amblyopia using fNIRS. While a growing body of evidence from RCTs and meta-analyses supports the efficacy of acupuncture for amblyopia,^[7–10]^ the evidence for Tuina remains largely anecdotal, relying on clinical experience and case series. This disparity underscores the rationale for our study: to preliminarily investigate the synergistic potential and neural correlates of this combined regimen, with a specific focus on the under-researched Tuina component. Based on our fNIRS findings and the observed clinical improvement, we propose a hierarchical modulation model of the visual pathway. This model posits that Tuina and acupuncture act synergistically at different levels: (1) At the peripheral level, Tuina is hypothesized to augment ocular blood flow, alleviate ciliary muscle spasm, and enhance retinal image fidelity,^[11]^ thereby optimizing the quality of sensory input to the visual cortex, a principle aligned with active vision therapy.^[12]^ (2) At the central level, acupuncture, particularly at vision-related acupoints such as BL60 along the Gallbladder and Bladder meridians, is postulated to regulate cortical excitability. Our observation of increased HbO in the primary visual cortex is consistent with prior fMRI research demonstrating that acupuncture at BL60 selectively activates this region.^[13]^ (3) The integration of these modalities may synergistically promote experience-dependent neuroplasticity,^[14]^ wherein improved peripheral input (via Tuina) creates a permissive environment that enhances the brain’s responsiveness to central neuromodulation (via acupuncture). The clinical outcome in this case is notable. The patient, who had exhibited a prolonged therapeutic plateau (BCVA stable at 0.6 LogMAR over 10 months) despite conventional occlusion therapy, achieved a substantial improvement of 0.4 LogMAR following 10 sessions of combined therapy. The reversal of such a plateau is significant, as spontaneous recovery in refractory amblyopia is considered rare.^[15,16]^ Furthermore, the observed visual acuity gains (BCVA of OD stabilized at 0.1 LogMAR) were sustained for over 3 years post-intervention, suggesting the potential for lasting functional improvement. The fNIRS data provided objective corroboration for this functional recovery. We observed a progressive increase in HbO and a concomitant decrease in HbR in the ipsilateral visual cortex during and after the intervention. This hemodynamic pattern is a well-established correlate of enhanced neural activity and strengthened neurovascular coupling in the neuroimaging literature.^[17]^ Interestingly, the contralateral visual cortex showed minimal hemodynamic changes. We hypothesize that this interhemispheric asymmetry may reflect the patient’s right-hemisphere dominance for visuospatial processing,^[18,19]^ with amblyopia exerting a more pronounced impact on the dominant hemisphere. Several limitations must be acknowledged. Primarily, the single-case design and absence of a control group preclude definitive causal attribution of the improvements to the intervention. While the temporal association is compelling, factors such as spontaneous variation cannot be entirely ruled out. Additionally, fNIRS measures hemodynamic correlates rather than direct neural activity; thus, inferences about underlying cellular mechanisms remain speculative and warrant future investigation with multimodal imaging approaches. In conclusion, this case report provides preliminary, hypothesis-generating evidence that the combination of acupuncture and Tuina may facilitate visual recovery in refractory amblyopia, potentially through synergistic modulation of the visual pathway. The objective fNIRS data and sustained long-term outcome strengthen the plausibility of a therapeutic effect. Future RCTs with sham-intervention control arms are essential to isolate the specific effects of each modality, validate this combined approach, and elucidate its precise mechanisms of action.

## 6. Patient perspective

After 10 sessions acupuncture and Tuina therapy, the patient shared his perspective on the treatment he received. He said, “After acupuncture and Tuina therapy, my vision improved steadily and my quality of life greatly improved, which increased my confidence in acupuncture and Tuina therapy.” Although formal developmental assessments were not performed, parental reports and school records indicated age-appropriate cognitive development. The child exhibited mild treatment-related anxiety during initial sessions that resolved with repeated exposure.

## 7. Conclusion

This single-case study provides preliminary evidence for the potential feasibility of combining acupuncture and Tuina therapy for amblyopia. The observed functional and neurophysiological improvements, as measured by visual acuity tests and fNIRS, are promising. This study also underscores the utility of portable fNIRS as an objective monitoring tool in such interventions.

However, the findings are constrained by significant methodological limitations. The single-case design inherently lacks a control group, preventing definitive causal attribution of the improvements to the intervention itself. Factors such as spontaneous variation, heightened attention in a research setting, or placebo effects remain plausible alternative explanations. Furthermore, while parental logs provided adherence metrics, the absence of electronic monitoring (e.g., for occlusion therapy) means adherence data may be subject to reporting bias.

Therefore, these results must be interpreted as preliminary and hypothesis-generating. They primarily highlight the necessity for future rigorous investigation. Subsequent research should employ randomized controlled trials (RCTs) with sham-acupuncture and/or sham-Tuina control arms to isolate the specific therapeutic effects from nonfactors and to validate the efficacy of this combined approach.

## Author contributions

**Data curation:** Xinyu Zhao, Ailing Bi, Hui Wang, Xiaotong Wu.

**Funding acquisition:** Xiuzhen Lu, Jianfeng Wu, Hongsheng Bi.

**Project administration:** Xiuzhen Lu, Hongsheng Bi.

**Supervision:** Hongsheng Bi.

**Writing – original draft:** Xinyu Zhao, Hui Wang, Xiaotong Wu.

**Writing – review & editing:** Ailing Bi, Xiuzhen Lu, Jianfeng Wu, Hongsheng Bi.
